# Improved Thermo-Hydraulic Stability and Boiling Heat Transfer Through a Novel Three-Layer Microchannel Heat Sink with 3/4 Open-Ring Pin Fin Arrays

**DOI:** 10.3390/ma19102143

**Published:** 2026-05-20

**Authors:** Guangyao Liu, Can Ji, Zhigang Liu, Peter D. Lund, Yeyao Liu, Fuqiang Xu, Shenglong Zhang, Cong Wang, Donghao Li

**Affiliations:** 1 Energy Research Institute, Qilu University of Technology (Shandong Academy of Sciences), Jinan 250014, China; lgy10281123@163.com (G.L.);; 2 School of Science, Aalto University, Puumiehenkuja 2, P.O. Box 15100, FI-00076 Espoo, Finland; peter.lund@aalto.fi

**Keywords:** three-layer microchannel, micro pin fin, flow boiling, hydrophilic surface, heat transfer enhancement

## Abstract

**Highlights:**

**What are the main findings?**

- Novel three-layer microchannels improve thermo-hydraulic stability and heat transfer;
- The thermo-hydraulic performance of the heat sink was investigated;
- A higher flow rate shifted boiling from a confined slug to a dispersed bubble flow;
- The average pressure drop was reduced by about 33% via hydrophilic treatment;
- The heat transfer coefficient was further enhanced by hydrophilic treatment.

**What are the implications of the main findings?**

- The new heat sink effectively improves overall thermo-hydraulic stability;
- Hydrophilic walls suppress vapor films and mitigate localized thermal accumulation;
- Thermal management viability for the low-carbon metallurgical process is enhanced.

**Abstract:**

This study systematically investigated flow boiling characteristics within a novel three-layer microchannel heat sink with 3/4 open-ring pin fin arrays, designed for high-heat-flux thermal management of low-carbon metallurgical reactors. Two-phase flow regimes, pressure drop, and wall temperature responses were analyzed. To evaluate the impact of functional surface material properties on thermo-hydraulic behavior, a hydrophilic nano-coating modification was applied to the inner copper channel walls for comparison. Increasing the flow rate triggered a transition from a vapor-dominated confined slug flow to a liquid-dominated dispersed bubble flow, which effectively improved the thermo-hydraulic stability. Hydrophilic surface modification resulted in an average pressure drop reduction of 33% and significantly diminished the sensitivity of flow resistance to velocity variations. Through hydrophilic treatment, the localized vapor film effect at high velocities was suppressed, and temperature field homogenization was promoted, yielding a maximum convective heat transfer coefficient of 7760 W/(m^2^·°C), i.e., 72.9% enhancement over the baseline heat sink. The underlying mechanism is attributed to the formation of a stable near-wall thin liquid film and the promotion of high-frequency nucleate boiling. These results will be of high relevance for developing efficient cooling solutions for power electronics, thereby supporting the advancement of low-carbon metallurgical reactors.

## 1. Introduction

Microchannel-based liquid cooling is widely recognized as a premier strategy for managing surging heat fluxes in modern electronics [1,2,3]. Meanwhile, low-carbon metallurgical processes such as hydrogen-based direct reduction are attracting increasing attention due to their potential for reducing CO_2_ emissions in the steel industry [4]. In these electrified metallurgical systems, high-power electronic converters and power supplies often face severe thermal challenges, creating an urgent need for more efficient cooling solutions. Conventional single-layer straight microchannels, despite their cooling potential, often suffer from pronounced axial temperature gradients and localized hot spots [5]. Simply increasing flow rates to mitigate these issues typically incurs excessive pumping power, creating a difficult design trade-off. Consequently, there is a strong demand for microchannel architectures that exploit three-dimensional (3D) flow organization and internal microstructures to simultaneously enhance heat transfer, improve temperature uniformity, and limit hydraulic losses [6].

From a structural standpoint, microchannel cooling research for high-heat-flux applications has largely followed two complementary routes. The first route focuses on multi-layer or stacked microchannel heat sinks, where several layers of channels are arranged in the vertical direction to expand the effective heat transfer area and reduce axial temperature gradients [7,8,9,10,11]. For example, two-layer or multi-layer configurations with parallel flow, counter flow or cross-flow arrangements have been shown to decrease overall thermal resistance and improve temperature uniformity at a given pumping power compared with a single-layer baseline [10,11,12,13,14]. Parametric and optimization studies further reveal that the geometric design of the channel height and width, fin thickness, and layer spacing of each layer strongly influences the distribution of heat load and flow among layers, which in turn affects the global thermo-hydraulic performance [11,12]. In the context of 3D-stacked chips, similar ideas underpin interlayer microchannel networks and manifold microchannel architectures, where multiple tiers and coolant layers are designed together as a 3D cooling system [7,8,9]. However, most existing stacked or multi-layer microchannel designs still employ simple straight channels with smooth or mildly finned rectangular cross-sections, and layer-to-layer differences are often limited to channel height or flow direction. The potential of combining multi-layer arrangements with more sophisticated internal microstructures to enable true 3D flow and heat transfer control remains underexploited.

The second major route for high-heat-flux removal is inserting micro pin fins or other protruding structures into the channel to disturb the boundary layer and promote fluid mixing [15,16,17,18]. Classical micro pin fin heat sinks use arrays of cylindrical or square pins distributed across the flow passage, and a large number of studies have mapped the influence of pin height, longitudinal and transverse pitch, and staggered versus in-line arrangements on both heat transfer and pressure drop [16]. Beyond conventional shapes, subsequent research includes diamond-shaped, streamlined, elliptical and compound pin fin geometries in pursuit of higher Nusselt numbers at a tolerable frictional loss [15,17,18]. These studies show that sharper-edged or more streamlined pins can either enhance vortex shedding and mixing or reduce local drag, and that the detailed pin shape has a pronounced effect on the onset and stability of flow boiling in two-phase operation [15]. Recent reviews conclude that pin fin configurations remain one of the most broadly applicable microstructural tools for single-phase and two-phase thermal enhancement in microchannels [6,18].

Open-ring-type micro pin fins have been proposed as a promising candidate to further refine the balance between heat transfer enhancement and pressure drop. In such geometries, each pin fin includes an internal cavity or a partially open annulus, creating local by-pass flow paths and complex recirculation zones. Deng et al. examined open-ring pin fin microchannels under flow boiling conditions and reported earlier boiling incipience, higher heat transfer coefficients and improved two-phase flow stability compared with several conventional pin shapes [19]. Zeng et al. integrated open-ring pin fins into a microchannel heat sink and highlighted that the periodic separation and recombination of flow around the open-ring structure significantly intensifies mixing and boundary-layer redevelopment, albeit at the cost of an increased pressure drop in single-phase operation [20]. Numerical simulations of non-closed ring-shaped pin fin arrays showed that carefully choosing the opening angle and spatial arrangement can maintain high Nusselt numbers while alleviating part of the excessive local drag [21]. A broader survey of pin fin applications confirms that open-ring microstructures offer attractive opportunities for tailoring local flow and heat transfer behavior [22] but emphasizes that most of these designs are still tested in idealized single-layer configurations, with limited experimental data and almost no work considering their role inside multi-layer, 3D-stacked cooling architectures.

As a result, the coupling between the 3D stacking strategy (number and arrangement of coolant layers) and the detailed in-channel microstructure (pin fin shapes, partial openings, 3D flow paths) remains only partially understood, and the potential benefits of combining multi-layer cooling networks with advanced micro pin fin geometries have not been fully quantified for realistic high-heat-flux conditions.

Overall, prior work on stacked or multi-layer microchannel heat sinks and on novel pin fin microstructures reveals several gaps. Firstly, most multi-layer or stacked microchannel configurations still rely on smooth or simply finned rectangular channels, with each layer possessing a relatively uniform and planar topology [7,8,9,10,11,12,13,14]. Flow and heat transfer enhancement is therefore dominated by macroscopic features such as the number of layers, flow direction (parallel, counter or crossflow), and the partitioning of coolant between layers, while the rich design space offered by complex 3D microstructures is scarcely explored. Secondly, studies on advanced pin fin shapes, especially open-ring-type geometries, are predominantly carried out in single-layer channels or cold plates, and their role in redistributing coolant among stacked layers, equalizing wall temperature across multiple tiers, and stabilizing two-phase flow in a 3D context has not been systematically evaluated [19,20,21,22]. Thirdly, existing ring-type designs often employ fully closed rings or large opening angles; these configurations can provide strong thermal enhancement but tend to incur substantial pressure losses and may pose fabrication challenges when scaled to wafer-level 3D integration, and only preliminary numerical work has considered partially open or short-arc ring geometries [21,22].

**Figure 1:**
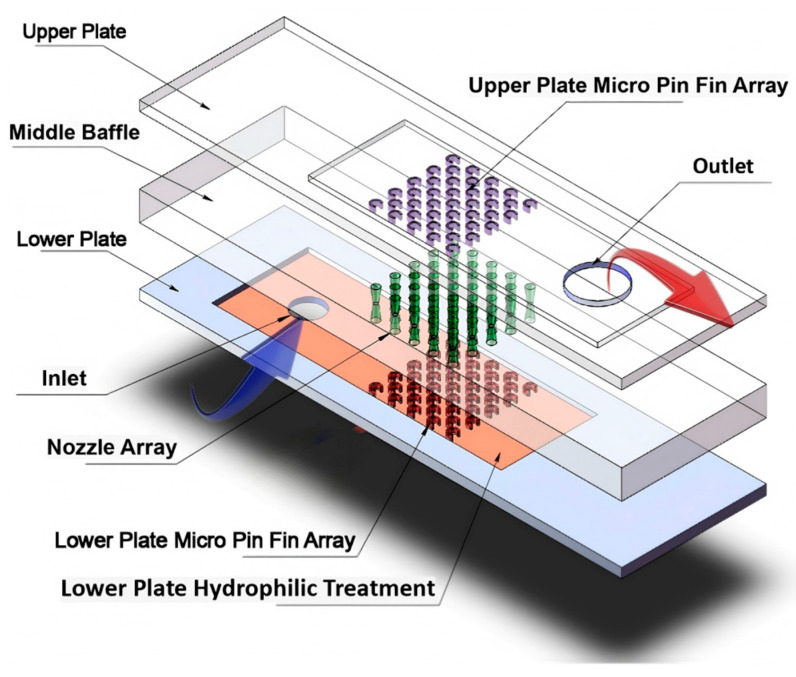
Exploded view of the three-layer microchannel heat sink.

To overcome the above challenges, a novel three-layer microchannel heat sink is proposed here, as shown in Figure 1, where the bottom and top layers are microchannels with 3/4 open-ring micro pin fin arrays, connected by a partition with an array of micro-scale convergent nozzles in the middle. This advanced hybrid micro pin fin heat sink features a significantly expanded heat transfer area and, intrinsically, the three-dimensionality of flow paths that blend streamwise, transverse and circumferential motion. This innovative design is expected to enable a synergistic enhancement in flow boiling heat transfer by combining the microstructure with surface modification. From an application perspective, such thermo-hydraulic enhancement strategies are particularly relevant for thermal management systems in low-carbon metallurgical reactors and high-power electrified process equipment, where compactness, operational stability, and high-heat-flux dissipation capability are critically required. In this study, the flow boiling heat transfer mechanisms within the three-layer microchannel structure were systematically investigated. Moreover, hydrophilic surface modification was applied to the inner channel walls to analyze the pressure drop characteristics and heat transfer performance before and after modification.

## 2. Experimental Study

### 2.1. Test Section

**Figure 2:**
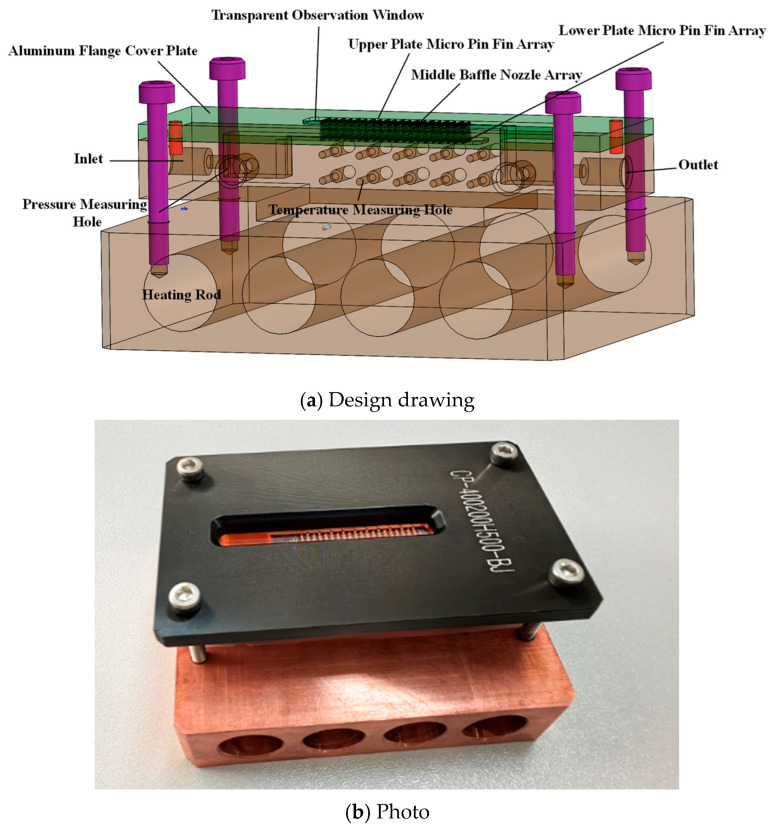
Three-layer microchannel heat sink test section.

**Figure 3:**
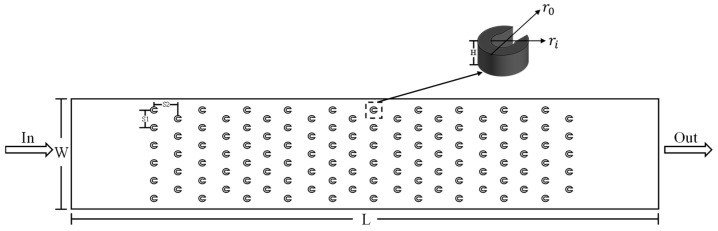
Schematic diagram of the top- and bottom-layer microchannels.

The test section was fabricated from copper via precision machining, with transparent glass plate on the top, and heating module at the bottom (as shown in Figure 2). Specifically, the microchannel and the micro pin fin arrays were manufactured using high-precision CNC micro-milling. The dimensional tolerance of the key geometric features was controlled within ±5 μm. The average surface roughness of the channel walls and pin fin surfaces was measured to be approximately 0.8–1.2 μm using a surface profilometer. The top packaging of the microchannel uses a Schott glass visualization cover, while the bottom is an integrated heating module with four cylindrical cutouts for inserting heating rods. The top and bottom layers of the microchannel are identical, as shown in Figure 3. The microchannel has a length, width, and height of 32 mm, 5.2 mm, and 0.5 mm, respectively. The micro pin fins adopt a three-quarter open circular ring configuration, with an inner diameter of 0.2 mm and an outer diameter of 0.4 mm. Each layer contains 110 micro pin fins arranged in 20 staggered rows. The detailed geometric parameters of the microchannel are summarized in Table 1. The inlet and outlet diameter of the nozzle array on the middle baffle corresponds to the inner and outer diameters of the micro pin fins, respectively.

### 2.2. Experimental System

**Figure 4:**
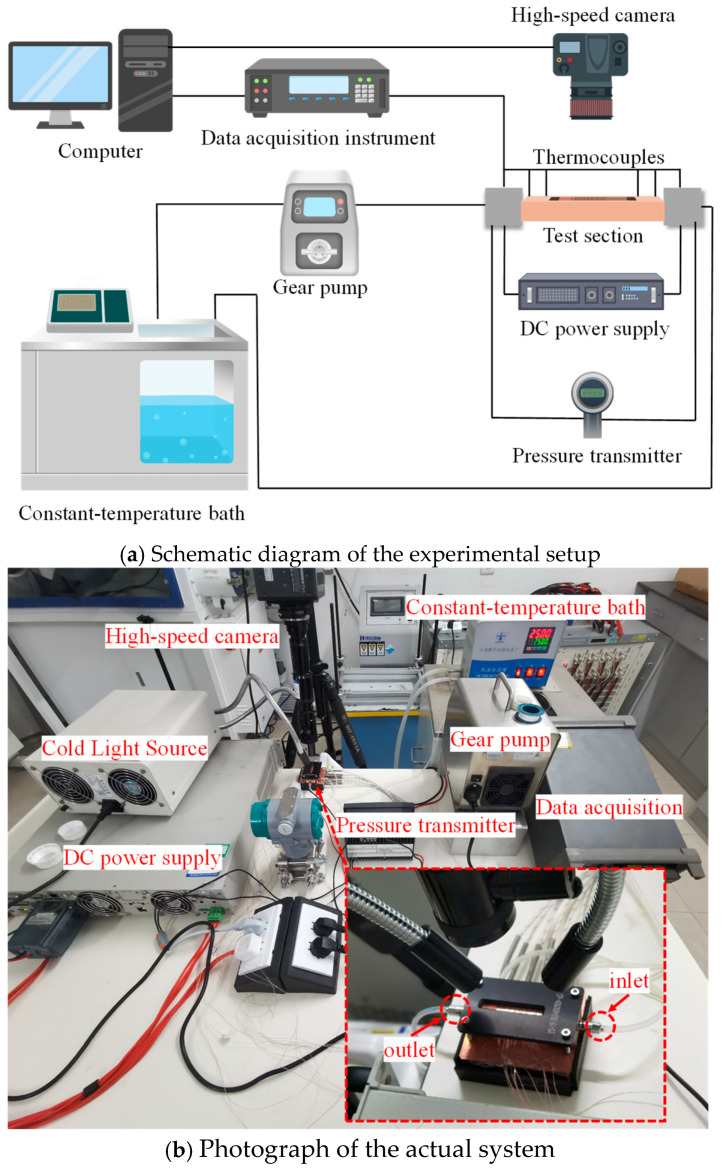
The experimental system.

The experimental system, shown in Figure 4, consists of four main parts: fluid circulation, heating, data acquisition, and visualization systems. The specifications and hardware used in the experimental system are listed in Table 2.

The working fluid is HFE7100 fluoride fluid (boiling point: 61 °C). The fluid flows into the micro pin fin channel from the non-open direction of the circular ring. The inlet flow rate of the test section was controlled by a gear pump. The working fluid temperature was regulated by heating the channel with an external heating bar, powered by a high-capacity programmable DC power supply. After passing through the test section, the fluid was cooled in a water storage tank and then returned to a constant-temperature bath. When the temperature of the microchannel bottom plate reached the set temperature, a cold light source was activated, and a high-speed camera recorded the boiling process within the microchannel pin fins array.

**Figure 5:**
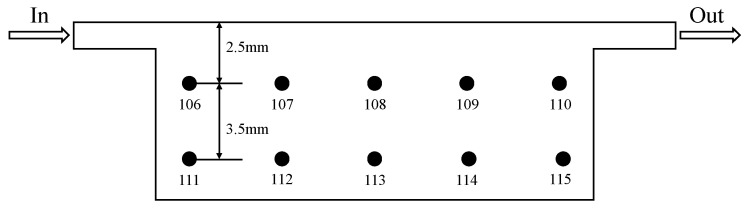
Temperature measuring hole diagram.

During the experiment, the pressure difference across the test section was measured by a differential pressure transmitter. Temperatures were measured with T-type copper-constantan thermocouples, and the signals were collected by a Fluke data acquisition instrument. The inlet temperature of the coolant (HFE7100, 3M Company, St. Paul, MN, USA) was maintained at 25 ± 0.5 °C throughout the experiments by the constant-temperature circulation system. To obtain the temperature at the bottom of the experimental section ($T_{surf}$), 10 temperature measuring holes, as shown in Figure 5, are arranged right below the microchannel with T-type thermocouples. The thermocouples were inserted horizontally from the side of the copper block and extended to positions directly underneath the centerline of the microchannel bottom wall. $T_{surf}$ can be calculated assuming one-dimensional thermal conductivity. The data measured by the data acquisition instrument were directly imported into the computer.

## 3. Data Processing and Error Analysis

### 3.1. Data Processing

The mathematical formulas used to process data are shown in Table 3.

### 3.2. Error Analysis

Based on the uncertainty propagation, Equation (1) is used to calculate the maximum possible error of the experimental parameters. The relative error of measured and derived parameters is listed in Table 4.

(1) $$\delta_{Y}=\sqrt{{\left(\frac{\partial Y}{\partial x_{1}}\right)}^{2}{\partial x_{1}}^{2}+{\left(\frac{\partial Y}{\partial x_{2}}\right)}^{2}{\partial x_{2}}^{2}+\cdots +{\left(\frac{\partial Y}{\partial x_{n}}\right)}^{2}{\partial x_{n}}^{2}}$$

## 4. Results and Discussion

### 4.1. Flow and Heat Transfer Performance of the Three-Layer Microchannel

#### 4.1.1. Flow Pattern in the Three-Layer Microchannel

**Figure 6:**
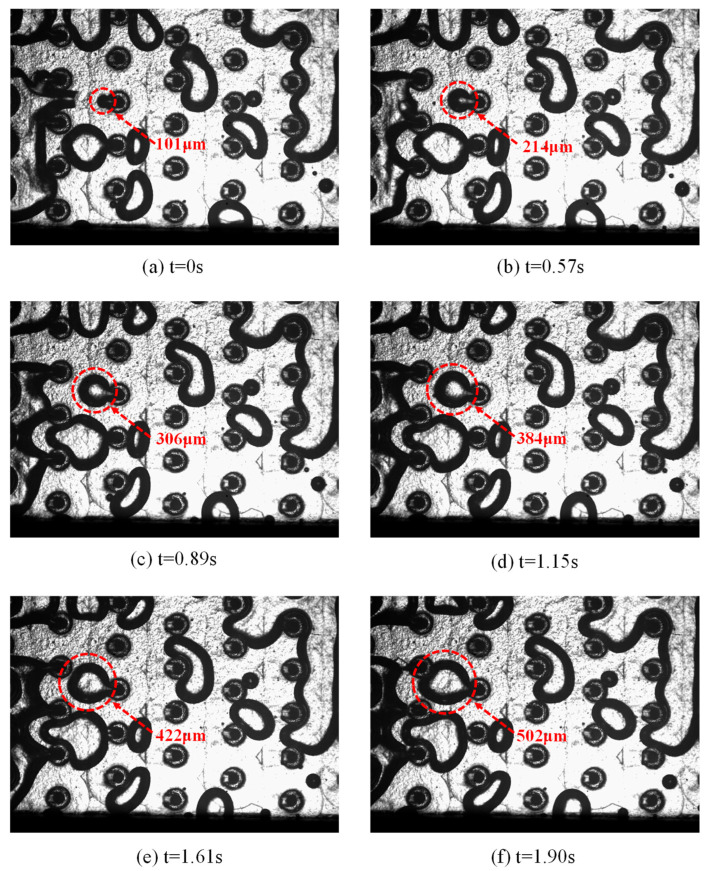
Temporal evolution of flow boiling patterns in the microchannel at a flow rate of 234.0 mL/min and a heating power of 124.8 W: (**a**) *t* = 0 s, (**b**) *t* = 0.57 s, (**c**) *t* = 0.89 s, (**d**) *t* = 1.15 s, (**e**) *t* = 1.61 s, and (**f**) *t* = 1.90 s.

Figure 6 illustrates the temporal evolution of two-phase flow patterns in the three-layer microchannel after the onset of boiling under a volumetric flow rate of 234.0 mL/min and a heating power of 124.8 W. Once nucleate boiling is initiated, discrete vapor bubbles are generated at active nucleation sites on the heated wall. At *t* = 0 s, small, isolated bubbles with a characteristic diameter of approximately 0.10 mm appear, indicating the initial stage of nucleate boiling.

With continuous heat input, vapor generation is intensified, and the bubbles grow rapidly due to evaporation at the liquid–vapor interface. At *t* = 0.57 s and *t* = 0.89 s, the bubble diameter increases to about 0.21 mm and 0.31 mm, respectively, and the bubbles exhibit obvious non-spherical morphology, mainly caused by the confinement effect of the microchannel and the shear force induced by the bulk liquid flow. Meanwhile, bubble departure and re-nucleation occur repeatedly, resulting in strong local flow disturbance.

As time further increases (*t* ≥ 1.15 s), bubble expansion becomes dominant, and neighboring bubbles tend to merge, forming larger vapor structures that partially block the channel cross-section. At *t* = 1.90 s, the characteristic bubble size reaches approximately 0.50 mm, and the flow pattern gradually transitions from dispersed bubbly flow to confined slug-like flow. This transition significantly enhances the liquid–vapor interaction and promotes liquid renewal near the heated surface, which is favorable for heat transfer enhancement. However, the increase in the vapor volume fraction also leads to higher flow resistance, implying a trade-off between heat transfer performance and pressure drop under high-heat-flux conditions.

**Figure 7:**
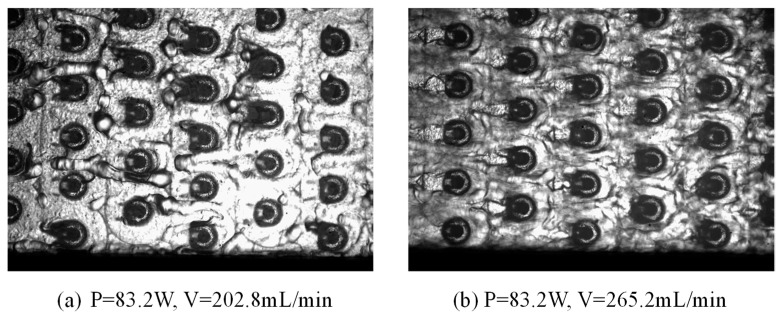
Stabilized two-phase flow patterns in the microchannel at a constant heating power of 83.2 W under different flow rates: (**a**) V = 202.8 mL/min and (**b**) V = 265.2 mL/min.

Figure 7 presents the stabilized two-phase flow patterns in the microchannel at a constant heating power of 83.2 W under two different volumetric flow rates, i.e., 202.8 mL/min and 265.2 mL/min. This flow rate range was selected to ensure the maintenance of a stable and continuous two-phase flow within the microchannel, avoiding significant flow oscillations or backflow that often occur at lower flow rates. Distinct flow regimes and bubble morphologies are observed, indicating that the flow rate plays a dominant role in regulating vapor accumulation and interfacial structures after the onset of boiling.

At the lower flow rate of 202.8 mL/min, vapor bubbles grow to relatively large sizes and exhibit obvious stretching and irregular shape, forming confined elongated bubbles or intermittent slug-like structures. The vapor phase occupies a considerable portion of the channel cross-section, leading to partial blockage of the flow passage. Due to the limited liquid momentum, bubble detachment is suppressed, and coalescence between neighboring bubbles becomes more frequent, resulting in a longer residence time of vapor near the heated wall. This flow pattern is characterized by strong vapor confinement and intermittent liquid replenishment, which may induce localized liquid film thinning and increase the risk of transient dry-out. In contrast, at the higher flow rate of 265.2 mL/min, bubbles remain smaller and are more uniformly dispersed along the channel, and the flow is dominated by dispersed bubbly or short-slug flow. The increased liquid inertia enhances bubble detachment and suppresses excessive bubble growth, thereby reducing vapor holdup and preventing the formation of long vapor plugs. Moreover, the stronger shear force and more frequent liquid renewal near the heated surface promote a more stable wetted condition, which is beneficial for maintaining sustained nucleate boiling and stable heat transfer.

Overall, under the same heating power, increasing the flow rate shifts the flow pattern from vapor-dominated confined structures toward more liquid-dominated dispersed bubbly flow. This transition effectively reduces vapor residence time and flow blockage, enhances liquid replenishment near the wall, and improves thermal–hydraulic stability in the microchannel. Therefore, the observed differences in flow morphology and vapor distribution provide a direct physical explanation for the variations in heat transfer performance and pressure drop characteristics under different flow rates, which will be further discussed in the following section.

#### 4.1.2. Pressure Drop

**Figure 8:**
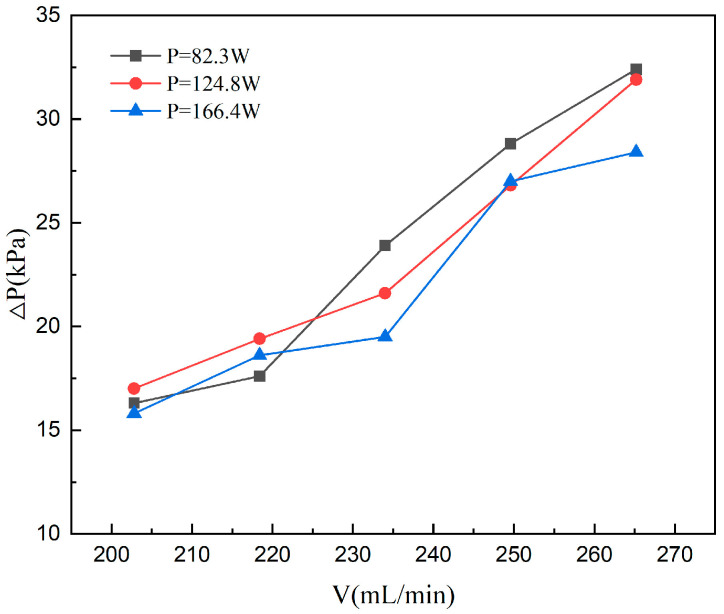
Variation of pressure drop with flow rate at different heating power.

Figure 8 illustrates the pressure drop (△*P*) characteristics within the heat sink under heating powers of 83.2 W, 124.8 W and 166.4 W as the flow rate increases from 202.8 to 265.2 mL/min. The heating power range is determined by both physical boiling criteria and operational considerations. The imposed heat flux needed to be sufficiently high to trigger the onset of nucleate boiling (ONB) within the present three-layer microchannel configuration, thereby ensuring the formation of stable and observable two-phase flow structures. Meanwhile, all operating conditions were maintained below the critical heat flux (CHF) safety limit to avoid severe dry-out, excessive thermal instability, or irreversible damage to the microchannel heat sink. The system pressure drop exhibits a significant upward trend with the increment of flow rate, which is primarily driven by the elevated Reynolds number and the subsequent increase in frictional resistance and local losses. In the low-flow-rate regime, the limited liquid supply causes the channel to be predominantly occupied by superheated vapor, leading to a typical film boiling state. As the flow rate further increases, a distinct transformation in the flow regime occurs, resulting in a sharp rise in pressure drop. This phenomenon is attributed to the flow-induced boiling mode transition from film boiling to nucleate boiling. During this transition, the density of active nucleation sites on the channel walls increases significantly. The continuous generation, growth, and detachment of bubbles complicate the two-phase flow patterns and intensify the local flow resistance, ultimately driving the total pressure drop to a maximum of 32.4 kPa.

Further comparison of the pressure drop characteristics under varying heating powers reveals that the △*P*-flow rate relationship for the 83.2 W and 124.8 W cases is approximately quasi-linear. In the low-flow-rate regime (202.8–218.4), the 124.8 W case exhibits a slightly higher △*P* than the 83.2 W case. At this stage, the reduction in fluid viscosity due to elevated wall temperatures is insufficient to dominate; instead, the synergistic effects of fluid property non-uniformity and variations in the entrance region length contribute to a marginal increase in flow resistance. As the flow rate reaches the intermediate stage (234.0), the △*P* for the 83.2 W case surpasses the others to become the highest. This shift indicates that at higher heat fluxes, the elevated mean fluid temperature significantly reduces viscosity, which partially offsets the resistance increment typically associated with increased flow rates. In contrast, the △*P* curve for the 166.4 W case remains consistently below the other two. Its growth rate markedly slows down in the intermediate regime, maintaining the lowest values or staying comparable to the moderate power case at high flow rates (249.6–265.2). This suggests that under substantial thermal loads, the pronounced temperature rise and further viscosity reduction lead to a significant decrease in the friction factor corresponding to a given volumetric flow rate, thereby dampening the sensitivity of pressure drop to flow rate variations.

In summary, at lower heating powers, the overall system pressure drop is relatively high due to weak boiling intensity and the dominance of single-phase flow resistance. Furthermore, the heating power influences the mean temperature and axial viscosity distribution, which in turn modulates the Reynolds number and the effective friction coefficient. Consequently, while the three curves share a consistent global trend, they exhibit distinct slopes and crossover behaviors, highlighting the significant impact of thermo-hydraulic coupling on the pressure drop characteristics within microchannels.

#### 4.1.3. The Bottom Temperature

**Figure 9:**
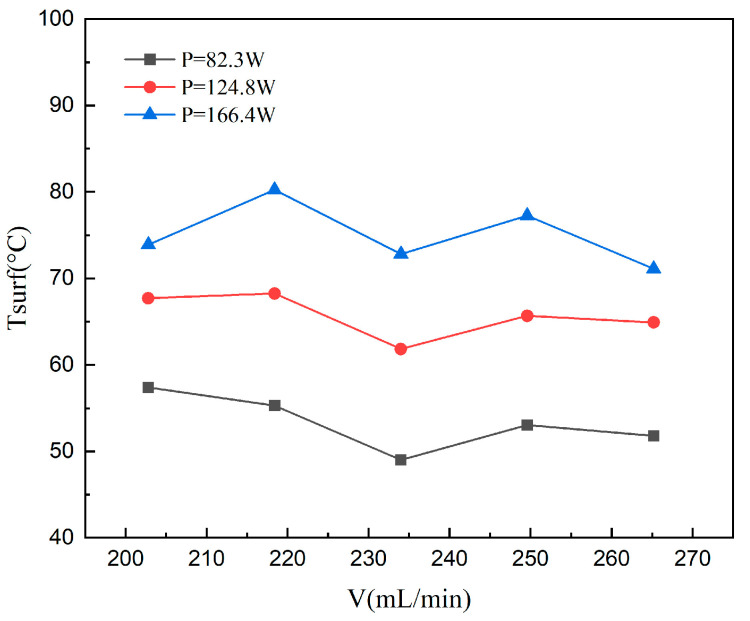
Variation of microchannel bottom temperature with flow rate at different heating power.

Figure 9 illustrates the variation of the average bottom surface temperature of the microchannel with the flow rate. Experimental data reveal that as the flow rate increases from 202.8 to 218.4 mL/min, the average temperature initially undergoes a slight elevation, subsequently declines, and eventually stabilizes after a gradual recovery. Within the low-flow-rate regime, the microchannel is characterized by vigorous film boiling. Despite the significant latent heat absorption capacity of the working fluid, the heat flux supplied by the heating rod exceeds the heat dissipated by the fluid. Consequently, localized heat accumulates in the near-wall region, leading to the observed initial rise in surface temperature.

**Figure 10:**
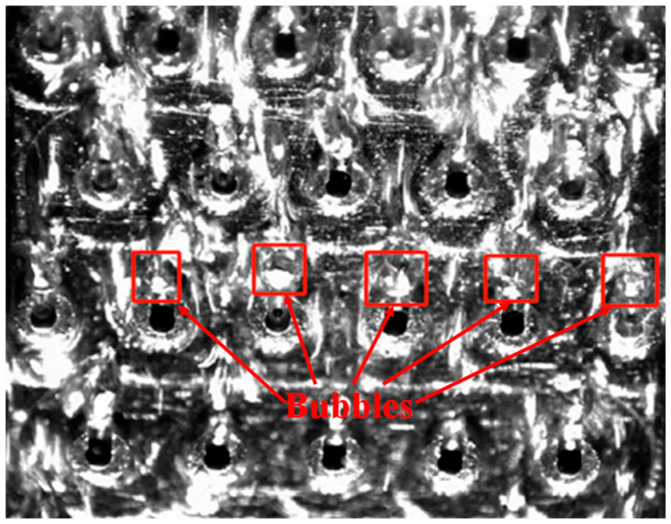
Transient distribution of microchannel bubbles under heating power of 83.2 W and flow rate of 249.6 mL/min.

Further increments in flow rate (218.4–234) strengthen convective cooling, causing the heat removal rate to surpass the input heat flux and resulting in a sharp drop in wall temperature. Conversely, a temperature recovery is observed at higher mass fluxes (234–265.2). This trend originates from bubble dynamics near the pin fins, where bubbles tend to accumulate in the open-ring gaps, as shown in Figure 10. Such bubble attachment diminishes the effective wetted area and introduces localized vapor film resistance, which impairs heat transfer performance. As the flow reaches a fully developed state with further velocity increases, the system approaches a new dynamic equilibrium, leading to a stabilized temperature profile.

**Figure 11:**
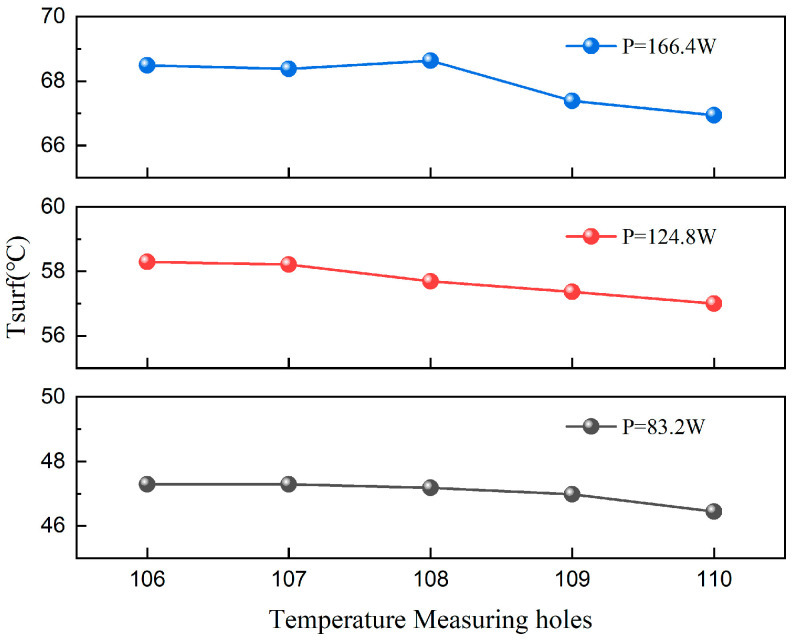
Streamwise temperature profile with the same flow rate at different heating power.

To further illustrate the bottom temperature distribution of the microchannel, the streamwise temperature profiles under different heating powers are compared at a moderate flow rate of 234 mL/min. As shown in Figure 11, data from five temperature measuring points (106–110) along the upper layer of the bottom surface are analyzed. Along the flow direction, the wall temperature exhibits a non-linear trend across all three heating powers, characterized by a stable upstream segment and a declining downstream segment. In the upstream and midstream regions (points 106–108), the surface temperature remains highly uniform with minimal fluctuation. Conversely, in the downstream region (points 108–110), the temperature paradoxically drops rather than rises, despite the continuous heat absorption by the fluid. This temperature reduction is particularly pronounced under the high-heat-flux condition of 166.4 W, where the local absolute temperature drop reaches 1.7 °C, approximately 2.1 times the drop observed in the 83.2 W case.

This downstream cooling phenomenon is primarily attributed to the flow pattern evolution during flow boiling and the consequent localized heat transfer enhancement. Corroborating the visual results presented in Figure 10 and previous sections, the fluid in the stable upstream region predominantly undergoes single-phase sensible heating or early-stage nucleate boiling. During this phase, nucleation sites are newly formed, and the bubble departure frequency is relatively low, which maintains a stable wall temperature. As the fluid advances downstream and continuously absorbs heat, the local vapor quality significantly increases, triggering a rapid transition in flow patterns. Within these high-quality flow regimes, the high-speed central vapor core exerts a strong interfacial shear force on the near-wall liquid film. This shear stress drastically thins the liquid film and substantially reduces thermal resistance, thereby enabling the heated wall to transfer heat to the fluid with significantly higher efficiency.

#### 4.1.4. The Convective Heat Transfer Coefficient

**Figure 12:**
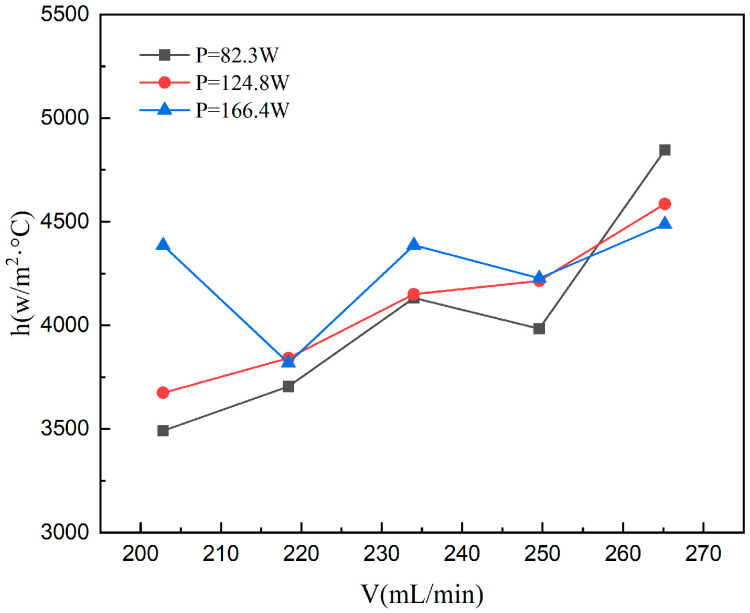
Variation of convective heat transfer coefficient with flow rate at different heating power.

The relationship between the convective heat transfer coefficient and flow rate at various heating powers is shown in Figure 12. The observed three-stage nonlinear trend of the heat transfer coefficient aligns well with the temperature variations reported in the preceding section. Notably, the divergent velocity-dependent behaviors of the heat transfer coefficient at higher power levels highlight a clear coupling between fluid flow and heat transfer.

At a heating power of 83.2 W, the convective heat transfer coefficient (*h*) exhibits a non-monotonic trend across three distinct flow rate regimes. In the low-flow-rate range (202.8–234), h increases consistently with the flow rate. This is primarily attributed to a transition from film boiling to nucleate boiling; the enhanced fluid disturbance destabilizes and ruptures the superheated vapor film on the wall. The subsequent increase in bubble nucleation density and departure frequency intensifies two-phase turbulence and near-wall mixing, leading to the observed augmentation of *h*. As the flow rate increases further (234–249.6), a critical reversal in h occurs. In this regime, rapid bubble nucleation and coalescence result in the formation of a stable, continuous vapor film covering the heated base and micro pin fin surfaces. This vapor layer acts as an additional thermal resistance, creating an insulation effect that offsets the convective enhancement provided by the increased flow rate. Consequently, the overall heat transfer performance degrades. In the high-flow-rate region (249.6–265.2), *h* recovers and shows an upward trend once more. Under these conditions, significantly enhanced shear forces reduce bubble adhesion and shorten the cycles of bubble growth and migration, hindering the formation of a stable vapor film. The high shear stress effectively strips and entrains the vapor into the bulk flow, allowing the liquid phase to re-wet the heating surface. This restoration of nucleate boiling as the dominant mechanism ultimately leads to the rebound of the heat transfer coefficient.

At 124.8 W, the convective heat transfer coefficient shows a smooth, monotonic increment with flow rate. Despite the increased impact of temperature-dependent fluid properties—driven by higher thermal levels than the 83.2 W case—the process is still governed by single-phase forced convection. The suppression of recirculation zones and heightened turbulence at higher velocities improve the predictability of the heat transfer performance, yielding a more regular trend.

At 166.4 W, the convective heat transfer coefficient exhibits a non-monotonic relationship with flow rate, reaching a local peak at 202.8 mL/min before undergoing a series of fluctuations. This behavior is attributed to the high heat flux, which increases the wall-to-fluid temperature difference and triggers complex thermo-hydraulic phenomena. Factors such as spatial variations in fluid properties, secondary flow evolution, and enhanced flow instabilities during boiling collectively counteract the expected enhancement from increased flow rate. As a result, the curve displays distinct non-monotonic and fluctuating characteristics instead of a simple linear or monotonic increase.

Comparison across different flow rates demonstrates that increasing heating power typically leads to an overall improvement in *h*, validating the heat flux removal capability of the novel three-layer microchannel design. Notably, at 265.2 mL/min, the heat transfer performance at 83.2 W marginally outperforms that at higher heat loads. This suggests that at high *Re*, further escalation of power triggers thermal maldistribution and increased local thermal resistance, which counteract the enhancement effects. These findings point to the existence of an optimal matching range between flow rate and thermal load for maximizing cooling efficiency.

Sensitivity analysis shows that the modulation of *h* by flow rate is inversely related to the heating power. The peak-to-valley fluctuation range decreases from ~40% at 83.2 W to ~20% at 166.4 W, suggesting reduced velocity sensitivity at higher heat fluxes. This transition implies that as the thermal load increases, the temperature-field-driven effects begin to outweigh the flow-driven enhancement, leading to a flatter curve with more pronounced local instabilities. Consequently, a systematic increase in *h* with flow rate is observed only at lower power levels, whereas high-power conditions are characterized by complex, non-monotonic fluctuations resulting from intense thermo-hydraulic coupling.

### 4.2. Effect of Surface Wettability on Hydraulic and Thermal Performance

Based on the preceding experiments, the effect of surface wettability on the performance of the three-layer microchannel heat sink was further investigated. Given that hydrophilic surfaces facilitate bubble departure and enhance liquid replenishment compared to hydrophobic counterparts [23,24], a hydrophilic nano-coating was applied to the microchannel’s internal surfaces. The coating is an alcohol–water-based transparent nanofluid primarily composed of anatase TiO_2_ nanoparticles with a solid content of 3–5 wt.%. According to the manufacturer’s technical specifications and related nano-TiO_2_ self-cleaning coating data, the average nanoparticle diameter is approximately 10–20 nm, and the water contact angle after coating treatment is lower than 5°. After deposition and room-temperature curing, a dense nano-scale hydrophilic thin film with an estimated thickness of approximately 80–150 nm is formed on the microchannel walls. Owing to the nanoscale thickness of the deposited layer, its influence on the original geometric dimensions of the microchannel and pin fin structures can be neglected. This alcohol–water-based solution creates a stable, nanometer-scale film that significantly lowers the contact angle, promoting the formation of a continuous liquid film through rapid spreading.

#### 4.2.1. Pressure Drop

**Figure 13:**
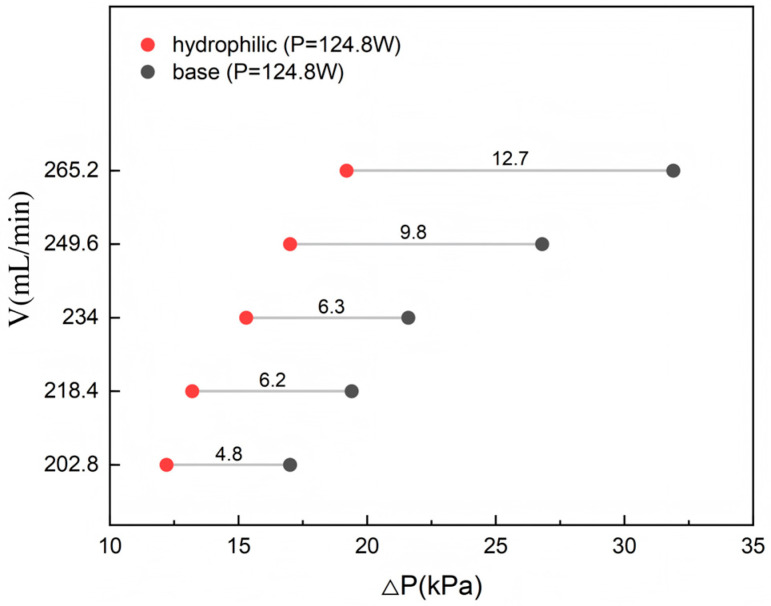
Comparison of the variation of pressure drop with flow rate at the same heating power.

The pressure drop (△*P*) for both baseline and hydrophilic microchannels at 124.8 W is presented in Figure 13. Although △*P* increases with flow rate in both cases, the distinct differences in their absolute values and growth rates underscore the critical role of surface wettability in modulating microscale flow resistance. From a quantitative perspective, the hydrophilic microchannels consistently exhibit a lower pressure drop compared to the baseline case, with the benefit becoming more pronounced at higher flow rates. While the pressure drop of the baseline case escalates by 88% (17.0 to 31.9 kPa) within the tested range, the hydrophilic channel shows a more moderate increase of 57% (12.2 to 19.2 kPa), representing an average 33% reduction in flow resistance. Notably, the linear fit of the △*P* − V relationship shows that the slope of the pressure drop curve of the hydrophilic channel (~0.11 kPa/(mL·min^−1^)) is roughly half that of the baseline case (~0.24 kPa/(mL·min^−1^)). These results demonstrate that hydrophilic modification effectively decouples the flow resistance from high-velocity penalties, providing a promising solution for energy-efficient thermal management in high-power-density applications.

The underlying mechanism for pressure drop reduction lies in the transition of the flow regime triggered by surface modification. On untreated surfaces, large contact angles induce localized vortices and flow separation, causing significant instability. Hydrophilic treatment promotes the formation of a stable, continuous liquid film on the channel walls. This film acts as a buffer that inhibits boundary layer separation, leading to a more ordered flow structure. Specifically, the treatment reduces the interfacial viscous resistance by lowering the contact angle and optimizes the near-wall velocity distribution, which decreases the Darcy friction factor. These synergistic effects explain why the hydrophilic microchannels exhibit superior hydraulic performance, maintaining a lower pressure drop even as the flow rate scales up.

#### 4.2.2. The Bottom Temperature

**Figure 14:**
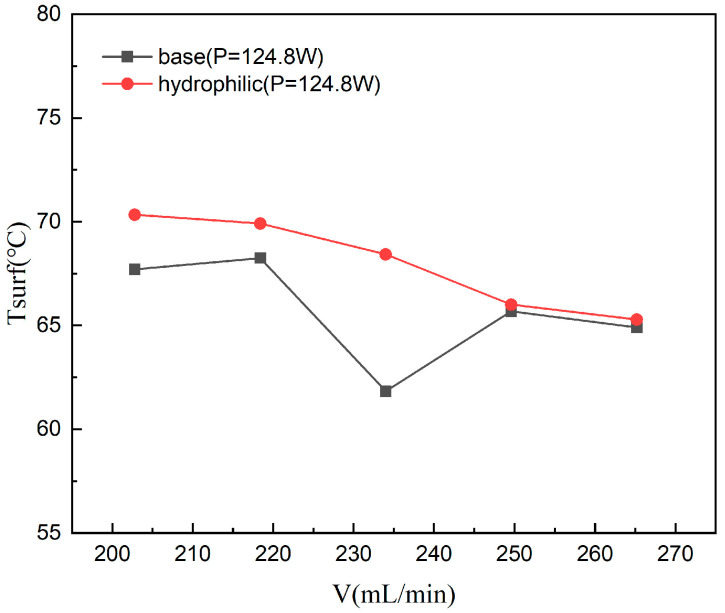
Comparison of the variation of microchannel bottom temperature with flow rate at the same heating power.

Figure 14 illustrates the average bottom surface temperature versus flow rate for both the unmodified baseline and the hydrophilic-treated microchannels at a constant heat load of 124.8 W. Different thermal responses are observed between the two test sections, underscoring the influence of surface wettability on heat transfer characteristics and temperature distribution. The results show that the bottom temperature of the hydrophilic microchannel exhibits a monotonic decrease with increasing flow rate. In contrast, the baseline channel displays a non-monotonic trend, reaching a minimum of 61.83 °C at 234.0 mL/min before slightly rebounding at higher flow rates. This rebound suggests the presence of localized flow stagnation or thermal maldistribution within the baseline channel, whereas the hydrophilic modification ensures a more stable and controllable thermo-hydraulic environment. Quantitatively, the temperature reduction for the baseline and hydrophilic channels is 4.1% (from 67.70 to 64.91 °C) and 7.2% (from 70.34 to 65.29 °C), respectively. Notably, the temperature flow rate curve for the hydrophilic structure is approximately 1.8 times that of the baseline channel, indicating its heightened sensitivity to flow conditions and superior potential for heat transfer enhancement at high velocities.

**Figure 15:**
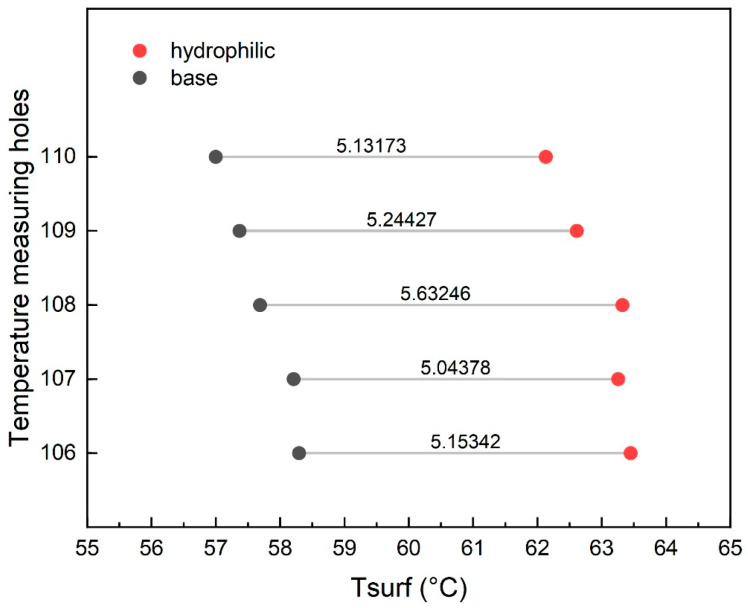
Streamwise temperature difference between hydrophilic and baseline microchannels under identical operating conditions.

**Figure 16:**
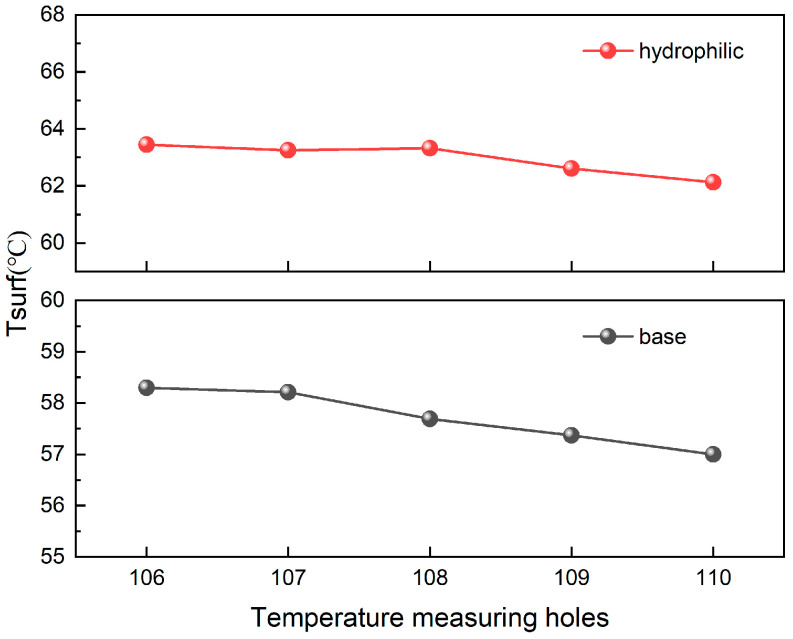
Comparison of streamwise temperature distributions trend in baseline and hydrophilic microchannels.

Further analysis was performed to clarify the temperature gap at 124.8 W and 234 mL/min by examining the streamwise thermal profiles. As shown in Figure 15, both channels exhibit a comparable temperature decline from the inlet to outlet (1.32 °C vs. 1.30 °C), suggesting a similar thermal development process. Nevertheless, the hydrophilic treatment leads to a systematic offset in temperature levels, with the streamwise temperature difference ranging between 5.04 and 5.63 °C (average 5.24 °C). This indicates that the thermal field modification is a comprehensive systemic effect rather than a localized coincidence. To better visualize this, Figure 16 displays the streamwise temperature distribution trend. It reveals that the temperature variation along the flow direction in the hydrophilic channel appears smoother, indicating the uniformity and continuity of its temperature field.

The underlying mechanism for these observations stems from the increased surface free energy and the reduced solid–liquid contact angle following hydrophilic treatment. These modifications elevate the threshold for gas film formation near the channel walls and effectively suppress localized dry-out. Consequently, enhanced liquid spreading and wettability facilitate a more uniform velocity distribution within the microchannels, which in turn mitigates localized vortices and flow stagnation zones. This homogenized flow condition ensures equitable heat removal from the heated walls, leading to a flatter and more uniform temperature field across the entire heat transfer surface. Due to this thermal homogenization effect, the average bottom surface temperature of the hydrophilic microchannels exhibits a smoother and more continuous response to increasing flow rate. Unlike the baseline case, it does not display the distinct multi-stage transition characteristics typically triggered by abrupt boiling regime changes or flow instabilities. In essence, by inhibiting non-uniform boiling and localized overheating, the hydrophilic treatment renders the system’s macroscopic thermal response a more linear and stable process.

In summary, hydrophilic modification of the three-layer microchannel heat sink achieves temperature field homogenization by optimizing fluid distribution and interfacial heat transfer within the microchannels. This treatment effectively dampens the drastic fluctuations in wall temperature induced by flow rate variations. These findings underscore that surface wettability engineering is a robust strategy for modulating micro-scale flow and heat transfer, thereby significantly enhancing the stability and reliability of thermal management systems.

#### 4.2.3. The Convective Heat Transfer Coefficient

**Figure 17:**
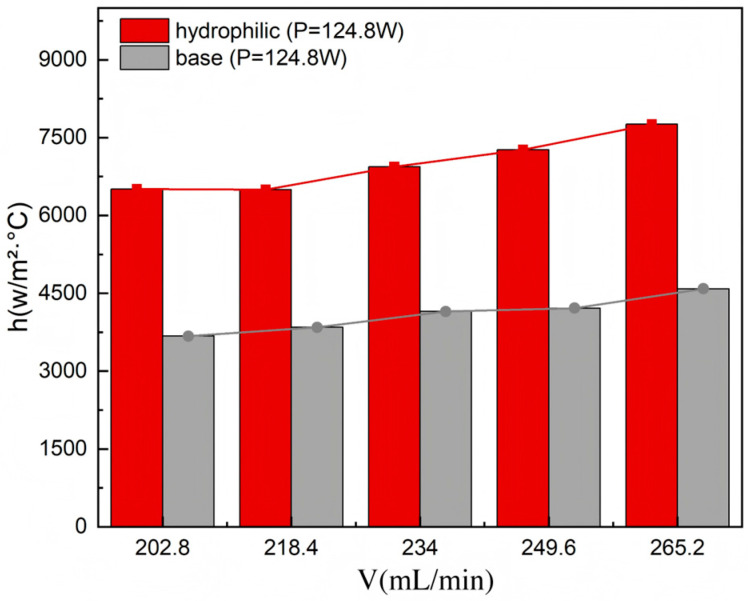
Comparison of convective heat transfer coefficient of the hydrophilic and the baseline microchannel heat sinks.

Figure 17 illustrates the quantitative relationship between the convective heat transfer coefficient (*h*) and the flow rate (ranging from 202.8 to 265.2 mL/min) under a constant heat load for different surface treatments. The experimental data clearly demonstrate that the evolution of *h* is highly dependent on the surface wettability characteristics of the microchannels. The hydrophilic modification significantly strengthens the solid–liquid interfacial interaction, thereby facilitating a more intensive and efficient thermal exchange process. Consequently, a peak convective heat transfer coefficient of 7760 W/(m^2^·°C) was achieved. Although the hydrophilic microchannel exhibits slightly higher measured surface temperatures under several operating conditions (Figure 14), this does not necessarily imply a lower convective heat transfer coefficient. According to Formula (10) in Table 3, the evaluated heat transfer coefficient depends not only on the superheat term, but also on the effective heat transfer rate. Owing to the enhanced wettability and improved liquid replenishment capability of the hydrophilic surface, the outlet fluid temperature increased noticeably, indicating more efficient heat absorption by the coolant. As a result, the apparent heat loss term calculated from the energy balance was significantly reduced compared with the baseline microchannel, and under several conditions exhibited a small negative deviation within the experimental uncertainty range. This reduction in effective heat loss compensated for the increase in wall temperature difference, ultimately resulting in a higher calculated convective heat transfer coefficient for the hydrophilic microchannel.

The underlying physical mechanism can be attributed to the fundamental optimization of the near-wall heat transfer process via surface modification. Specifically, the hydrophilic treatment substantially reduces the contact angle at the solid–liquid interface, facilitating the rapid spreading of the liquid phase into a thin, continuous film on the microchannel walls. This liquid layer not only effectively mitigates interfacial thermal resistance but also accentuates the onset of nucleate boiling (ONB) at the micro-scale. By promoting the inception and rapid departure of vapor embryos, the modification significantly elevates the phase-change heat transfer efficiency. In contrast, the untreated surface tends to induce unstable vapor blankets or oversized bubbles on the heating area, which act as an additional thermal barrier and hinder efficient heat dissipation.

Notably, during the third stage dominated by single-phase convection, the hydrophilic-treated microchannel yields a 72.9% increase in the maximum heat transfer coefficient compared to the baseline microchannel. This significant augmentation demonstrates the efficacy of surface-wetting for enhancing heat transfer across both single-phase and transition boiling regimes. The hydrophilic surface breaks the thermal performance bottleneck of traditional surfaces by facilitating stable thin-film evaporation and inducing earlier, more uniform nucleate boiling. These findings underscore the critical role of interfacial engineering in advancing high-performance micro-scale thermal management solutions.

In summary, hydrophilic treatment enhances interfacial wettability, thereby optimizing heat transfer pathways and improving thermal efficiency in microchannels. This modification enables stable and enhanced thermal performance across both single-phase and phase-change heat transfer regimes. The findings of this study provide experimental data support and theoretical guidance for the design and optimization of next-generation high-performance microchannel heat sinks.

## 5. Conclusions

This study investigated the flow boiling characteristics of a novel three-layer microchannel heat sink, featuring 3/4 open-ring pin fin arrays. Furthermore, hydrophilic surface modification was introduced to elucidate the influence of surface wettability on hydraulic and thermal performance. By analyzing the evolution of two-phase flow regimes, local vapor quality variations, pressure drop behavior, wall temperature responses, and convective heat transfer coefficients, this work revealed the complex multi-field coupling mechanisms inherent to the phase-change heat transfer process in this specific three-dimensional architecture. The main conclusions are as follows:

1. At a constant heat load, increasing the flow rate triggers a transition in the two-phase flow regime from vapor-dominated confined slug flow to liquid-dominated dispersed bubble flow. This transition effectively shortens vapor residence time and enhances near-wall recirculation, thereby improving the overall heat transfer stability;
2. Within the flow range of 202.8–265.2 mL/min, the two-phase system pressure (△*P*) is highly dependent on the interplay between operating conditions, flow regimes, and local vapor quality. While △*P* generally exhibits an upward trend with increasing flow rate due to elevated frictional resistance, this behavior is strongly modulated by the applied heat flux. At higher heating powers, the elevated mean fluid temperature leads to reduced viscosity and a lower friction factor, which diminishes the sensitivity of △*P* to flow rate variations. This highlights a pronounced thermo-hydraulic coupling effect within the microchannels;
3. The bottom surface temperature exhibits a non-monotonic variation with flow rate, reflecting the dynamic competition between flow regime transitions and localized thermal resistances, such as localized vapor accumulation, which are highly dependent on the local flow regime. The temperature undergoes a three-stage evolution: “slight rise—rapid decline—slow recovery and stabilization”;
4. The convective heat transfer coefficient (*h*) displays distinct three-stage nonlinear characteristics. Under low-power conditions, *h* follows a “rise—fall—rise” trend, which is attributed to shifts in boiling regimes involving early nucleate boiling followed by intensified bubble interaction and partial vapor coverage, eventually leading to the destabilization and subsequent breakup of the localized vapor layer. Medium-power conditions show a near-monotonic increase, whereas high-power conditions exhibit significant fluctuations in *h* due to the strong coupling of flow and thermal fields, suggesting a higher susceptibility to unstable responses under high heat fluxes;
5. Hydrophilic surface treatment significantly reduces system pressure drop and enhances flow stability. At 124.8 W, △*P* of the hydrophilic microchannel is 33% lower than that of the baseline, with the slope of the △*P* − V curve reduced by half;
6. Hydrophilic modification promotes a more uniform thermal field and improves the system’s thermal responsiveness to flow rate changes. The bottom surface temperature of the hydrophilic microchannel exhibits a monotonic decline, with a temperature drop 1.8 times that of the baseline microchannel;
7. Hydrophilic treatment substantially improves convective heat transfer capacity. A maximum of 72.9% enhancement in heat transfer coefficient was achieved over the baseline case. The underlying mechanism lies in the capacity of the hydrophilic surface to facilitate the maintenance of a favorable thin liquid film and to support conditions conducive to high-frequency, relatively consistent nucleate boiling. These effects contribute to the reduction of interfacial thermal resistance and the enhancement of phase-change efficiency within the investigated parameters.

## Figures and Tables

**Table 1:** Geometric parameters of the microchannel.

Geometry Variable	Value	Geometry Variable	Value
$r_{0}$	0.2 mm	*L*	32 mm
$r_{i}$	0.1 mm	*W*	5.2 mm
*H*	0.5 mm	$S_{1}$	0.8 mm
*n*	220	$S_{2}$	1.2 mm

**Table 2:** Specifications and models of equipment in the experimental system.

Equipment	Specifications and Models	Technique Parameters
DC power supply	ITECH IT6515D, ITECH, Nanjing, China	500 V/20 A/1800 W
Differential pressure transmitter	Rosemount3051, Rosemount, Shakopee, MN, USA	0–100 kPa
Intelligent gear pump	LeadFluidCT3000F, Lead Fluid, Baoding, China	15–2700 mL/min
Data acquisition instrument	Fluke 2638 A, Fluke, Everett, WA, USA	±0.0024%
Thermocouple	Omega TT-K-40-SLE, Omega, Norwalk, CT, USA	±0.1 °C
High-speed camera	Fastcam Mini AX100, Photron, Tokyo, Japan	50–212,500 fps
Cold light source	HDL-II, Betical, Suzhou, China	~220 ± 22 V 50 Hz

**Table 3:** Mathematical formulas for experimental data.

Parameter	Formula	No.
*Re*	$Re=\frac{u_{max}D}{v}$	(1)
Maximum velocity of flow	$u_{max}=\frac{V}{A_{min}}$	(2)
Equivalent diameter	$D=\frac{4A}{P_{s}}$	(3)
Flow resistance coefficient	$f=\frac{2∆P}{N_{x}\rho u_{max}^{2}}$	(4)
Average temperature of the lower layer	$T_{1}=\frac{T_{106}+T_{107}+T_{108}+T_{109}+T_{110}}{5}$	(5)
Average temperature of the upper layer	$T_{2}=\frac{T_{111}+T_{112}+T_{113}+T_{114}+T_{115}}{5}$	(6)
The bottom temperature	$T_{surf}=T_{1}-\frac{(T_{2}-T_{1})\times S_{1}}{S_{2}}$	(7)
Heat transfer area	$S=2WL-\frac{3}{4}n\pi \left(r_{0}^{2}-r_{i}^{2}\right)+nH\left[\frac{3}{2}\pi \left(r_{0}+r_{i}\right)+2\left(r_{0}-r_{i}\right)\right]+2n\pi \left(r_{0}+r_{i}\right)$	(8)
Total heat loss of flow heat exchange	$Q_{loss}=P-c_{P}V\rho (T_{out}-T_{in})$	(9)
The average heat transfer coefficient	$h=\frac{\left(P-Q_{loss}\right)}{S(T_{surf}-\frac{T_{in}+T_{out}}{2})}$	(10)
*Nu*	$Nu=\frac{hD}{\lambda }$	(11)

**Table 4:** Range and error of the main parameters.

△*D*/*D*	△*V*/*V*	△*Cp*/*Cp*	△*Nu*/*Nu*	△*h*/*h*	△λ /λ	△*P*/*P*
1.62	0.88	0.5	2.41	1.71	0.5	1.01

## Data Availability

The original contributions presented in this study are included in the article. Further inquiries can be directed to the corresponding authors.

## Nomenclature

$r_{0}$	outer diameter of the micro pin fin (mm)	Re	Reynolds number
$r_{i}$	inner diameter of the micro pin fin (mm)	Nu	Nusselt number
*H*	height of micro pin fins channel (mm)	*t*	time (s)
*n*	number of micro pin fins	*D*	equivalent diameter (m)
*L*	length of micro pin fins channel (mm)	*V*	volumetric flow rate (mL/min)
*W*	width of micro pin fin channel (mm)	$T_{in}$	the inlet temperature (°C)
$S_{1}$	horizontal spacing of micro pin fins (mm)	$T_{out}$	the outlet temperature (°C)
$S_{2}$	longitudinal spacing of micro pin fins (mm)	$T_{surf}$	the bottom temperature (°C)
∆P	pressure drop between inlet and outlet of micro pin fins channel (kPa)	$T_{i}$ (i = 1, 2, 3)	measuring hole temperature of microchannel (°C)
*P*	heating power of DC power supply (W)	*A*	cross-sectional area (m^2^)
S	heat transfer area (m^2^)	u	velocity of fluid (m/s)
h	the average heat transfer coefficient (W/(m^2^⋅K))	Subscript	
		0	benchmark
$Q_{loss}$	heat loss (kW/m^2^)	min	minimum
		max	maximum
